# Walruses on the Dnieper: new evidence for the intercontinental trade of Greenlandic ivory in the Middle Ages

**DOI:** 10.1098/rspb.2021.2773

**Published:** 2022-04-13

**Authors:** James H. Barrett, Natalia Khamaiko, Giada Ferrari, Angélica Cuevas, Catherine Kneale, Anne Karin Hufthammer, Albína Hulda Pálsdóttir, Bastiaan Star

**Affiliations:** ^1^ Department of Archaeology and Cultural History, NTNU Vitenskapsmuseet, Norwegian University of Science and Technology, 7491 Trondheim, Norway; ^2^ Institute of Archaeology, National Academy of Sciences of Ukraine, 12 Heroiv Stalingrada Ave., 04210 Kyiv, Ukraine; ^3^ Centre for Ecological and Evolutionary Synthesis, Department of Biosciences, University of Oslo, PO Box 1066, Blindern, 0316 Oslo, Norway; ^4^ McDonald Institute for Archaeological Research, Department of Archaeology, University of Cambridge, Downing Street, Cambridge CB2 3ER, UK; ^5^ Department of Natural History, The University Museum, University of Bergen, PO Box 7800, 5020 Bergen, Norway

**Keywords:** ecological globalization, ancient DNA, stable isotopes, historical ecology, Middle Ages, *Odobenus rosmarus rosmarus*

## Abstract

Mediaeval walrus hunting in Iceland and Greenland—driven by Western European demand for ivory and walrus hide ropes—has been identified as an important pre-modern example of ecological globalization. By contrast, the main origin of walrus ivory destined for eastern European markets, and then onward trade to Asia, is assumed to have been Arctic Russia. Here, we investigate the geographical origin of nine twelfth-century CE walrus specimens discovered in Kyiv, Ukraine—combining archaeological typology (based on *chaîne opératoire* assessment), ancient DNA (aDNA) and stable isotope analysis. We show that five of seven specimens tested using aDNA can be genetically assigned to a western Greenland origin. Moreover, six of the Kyiv rostra had been sculpted in a way typical of Greenlandic imports to Western Europe, and seven are tentatively consistent with a Greenland origin based on stable isotope analysis. Our results suggest that demand for the products of Norse Greenland's walrus hunt stretched not only to Western Europe but included Ukraine and, by implication given linked trade routes, also Russia, Byzantium and Asia. These observations illuminate the surprising scale of mediaeval ecological globalization and help explain the pressure this process exerted on distant wildlife populations and those who harvested them.

## 1. Introduction

**Figure 1.**
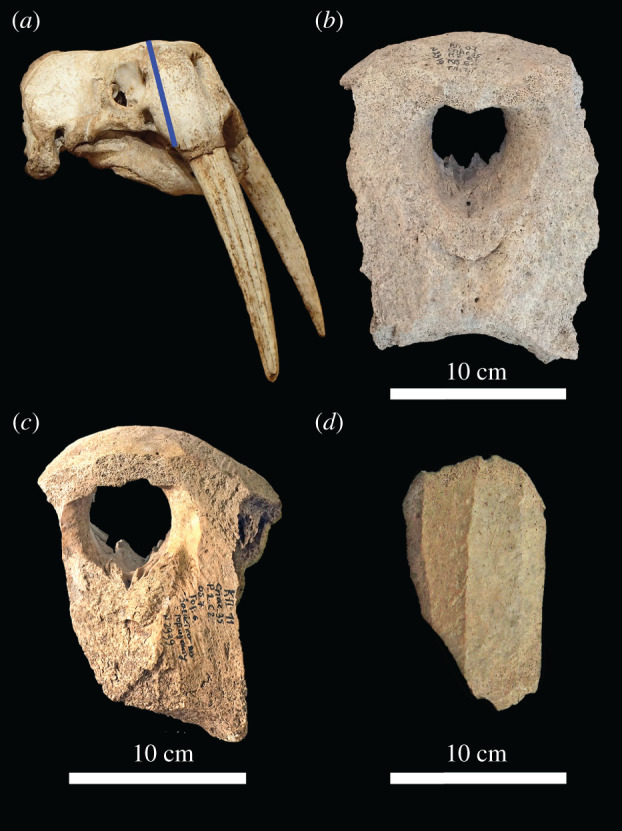
Walrus rostra from mediaeval Kyiv: (*a*) complete modern skull for comparison, with a line indicating the plane of rostrum removal; (*b*) anterior view of R64, showing breakage of the tusk sockets for ivory removal; (*c*) anterior view of R73, showing carving of the anterior surface and rough faceting of the surviving dorsal portion of the left tusk socket; (*d*) lateral view of R66, showing how the tusk socket was thinned by carving rough parallel facets. Scale bars *ca* 10 cm. Photographs: James H. Barrett and Natalia Khamaiko. (Online version in colour.)

The depletion of walruses by mediaeval hunting in Iceland and Greenland is an important pre-modern example of ecological globalization, a process by which increasingly distant rural or hunter/gatherer communities were linked with centres of consumption such as major cities [1–3]. European demand for walrus products was high during the Middle Ages, driven by the popularity of carved morse ivory among ecclesiastical and secular elites and of walrus-hide ropes for heavy lifting tasks [4–7]. Material evidence of the resulting trade survives as ivory artefacts and as pieces of modified walrus skulls (rostra) in which pairs of tusks were transported (figure 1). Studies of these rostra, using archaeological, ancient DNA (aDNA) and stable isotope methods, have demonstrated that most examples from Northwestern Europe were probably imported from Norse Greenland [1,3]. The walruses were hunted in western Greenland, and conceivably also in the eastern Canadian Arctic, with pairs of tusks in rostra then sent to centres such as Dublin, Trondheim, Schleswig and Bergen for carving and/or onward trade [3,8].

Centres in Eastern Europe also provide evidence of mediaeval walrus trade. Novgorod, Russia, has produced one of the largest collections of carved mediaeval walrus ivory in Europe [9,10] and excavations in Kyiv, Ukraine, revealed nine walrus rostra—more than most northern European centres; only Bergen and Schleswig, with 15 examples each, have more [3,11,12]. Moreover, mediaeval Arabic written sources regarding trade in Central and Western Asia refer to ‘fish teeth' interpreted as walrus tusks acquired in or via what are now Ukraine and Russia, valued for making knife handles and sword hilts [13–15]. Historical evidence from mediaeval Byzantium also suggests imports of walrus ivory from Ukraine or Russia [16]. It has long been posited that the most important source of walrus imports for mediaeval eastern Europe was Arctic Russia [5,6,9,17–19], but the origin of these finds is in fact hitherto unknown.

Archaeological descriptions of one rostrum from Novgorod and three from Kyiv have been published [3,9,11,12], but without aDNA and stable isotope analysis. Six additional Kyiv specimens were discovered by one of us (N.K.) during excavations and recent post-excavation analysis. Here, we report new aDNA and stable isotope analyses on seven of the nine Kyiv specimens (those available for sampling: R64, R65, R66, R71, R72, R73, R74) and provide an archaeological *chaîne opératoire* (operational sequence) assessment of the way in which the seven sufficiently complete examples (R64, R65, R66, R71, R72, R73, R76) were prepared for trade.

As a result, it is possible to solve the mystery of their likely source. An understanding of these rostra also casts light on the potential origins of the abundant walrus ivory from Novgorod, given that the latter city lies on the river trade route through which any source (Arctic Russian, Greenlandic or otherwise) would reach Kyiv. Finally, the Kyiv samples may illuminate the ultimate origins of tusks traded onward to Byzantium and Asia in the Middle Ages, as known from the Greek and Arabic written sources [13–16]; the city was an important nexus of trade, with especially strong links to Scandinavia, Germany, Poland and Constantinople [20–23]. Thus an understanding of ecological globalization—and of the potential scale of demand focused on Greenland's walruses—can be greatly enhanced by determining the ultimate origins of the Kyiv walrus finds.

## 2. Material and methods

### (a) Archaeology and zooarchaeology

The Kyiv walrus specimens all included part of at least one tusk socket, facilitating confident morphological identification. They had fused cranial sutures and are inferred to be from adult individuals. The specimens were excavated in 2007, 2008 and 2011, from Horizons 5 and 6 of the 35 Spaska Street site in the Podil district [20–22]. This part of the lower town was periodically flooded, creating a sequence of clear and closely dated (based on artefact types and superposition) cultural and alluvial layers. Given their find contexts, the rostra date to the mid-twelfth (R71, R72, R73, R74) and late-twelfth (R64, R65, R66, R75, R76) centuries.

Seven of the nine specimens preserved enough evidence of past modification to be classified according to a four-category *chaîne opératoire* typology established by Barrett *et al*. [3], based on the characteristics of all previously known mediaeval European finds of walrus rostra (see electronic supplementary material, figure S1). The types capture sets of increasingly elaborate steps used to remove and sculpt each rostrum; the modifications produced a compact decorated package from which the tusks could be easily removed when needed for ivory carving. The four-stage typology also represents a chronological sequence, starting in the eleventh century CE and ending (with the exception of residual outliers redeposited in later archaeological layers) in the fourteenth or fifteenth centuries CE.

Type 1 rostra were only modified by characteristic cut marks made during removal from the skull. In type 2 rostra, the tusk alveoli were thinned by rough parallel cuts that faceted the surface of each socket. Type 3 rostra have the characteristics of types 1 and 2, while also exhibiting decorative carving of the nasal aperture. Type 4 rostra have the characteristics of preceding types, but the tusk sockets are smoothly rather than roughly facetted, and the ventral margin between the tusks is invariably carved. Fragmentary rostra lacking some information can be attributed to broader categories (e.g. type 2/3).

To establish the type(s) of each Kyiv specimen, modifications were recorded under low magnification: how and from what direction the rostrum was cut from the skull; whether and how the posterior margins of the severed rostrum were finished; whether and how the nasal aperture was shaped; whether and how the anterior surface between the tusks was shaped; whether and how the tusk sockets were thinned to ease extraction of the tusks; whether and how the ventral margin between the tusks was shaped and whether the sockets were broken during tusk removal (electronic supplementary material, figure S1 and table S1).

Where practicable, the maximum and minimum cross-section of each tusk socket was measured as a proxy for animal size and sex (electronic supplementary material, table S2). Given dimorphism, Atlantic walruses with a maximum tusk socket cross-section of ≤44 mm are likely to be female, whereas those with cross-sections of ≥54 mm are probably male, a criterion corroborated by aDNA sexing [3]. Sex is relevant because males produce larger tusks and might thus have been preferred targets of hunting [3].

Samples of ≥1 g were cut from seven of the Kyiv rostra using a micromotor, stored in separate plastic bags and shipped to the University of Oslo aDNA laboratory. Here, a subsample of each was cut and sent to the University of Cambridge for stable isotope analysis.

### (b) aDNA

All laboratory work was conducted in a dedicated aDNA clean laboratory at the University of Oslo following standard anti-contamination and authentication protocols (e.g. [24,25]). Subsamples of the seven Kyiv rostra were UV-treated for 10 min per side and pulverized by stainless-steel mortar [26]. aDNA was extracted from 2 × 200 mg of bone using a mild bleach and double digestion (BLEDD) step [27,28]. Double-stranded sequencing libraries were prepared following the Meyer–Kircher protocol [29] with modifications [30] and were paired-end sequenced on an Illumina HiSeq4000 at the Norwegian Sequencing Centre.

Whole genome shotgun sequencing data from the seven Kyiv rostra were jointly analysed with genomic data from 37 walrus specimens obtained by Star *et al*. [1]. Reads were processed (PALEOMIX v. 1.2.14) [31], collapsed (AdapterRemoval v. 1.5) [32] and aligned (BWA *aln* v.0.7.5a-r405) [33] to the Pacific walrus genome assembly [34] and the Atlantic walrus mitogenome [35]. We used the chromosome resolved HiC genome assembly obtained from the DNA ZOO consortium [36]. We determined genetic sex for the Kyiv specimens (and several new stable isotope control samples noted below) by calculating the relative coverage of the X chromosome in comparison to autosomal walrus chromosomes (X:A coverage, following [3,37,38,39]). aDNA damage was assessed (mapDamage v. 2.0.6) [40]) excluding alignments with a threshold of MapQ-value < 25. Genotypes for the mitogenome were jointly called (GATK v. 4.1.4.0) [41], after duplicate removal (PicardTools v. 1.96), indel realignment (*IndelRealigner*), and filtered (BCFTOOLS v. 1.9) [42] *-i* ‘FS < 60.0 && SOR < 4 && MQ > 30.0 && QD > 2.0’, *–SnpGap* 10. Indels were excluded, and genotypes with quality <15 and read depth less than 3 were set as missing (VCFTOOLS v. 0.1.16) [43].

We analysed ∼43 million reads for the seven Kyiv rostra, yielding between 2% and 41% endogenous DNA and two- to 28-fold coverage of the mitogenome (electronic supplementary material, table S2). The Kyiv rostra are classified according to the two main mitogenomic branches that have been identified in Atlantic walrus: a western clade restricted to western Greenland and Eastern Arctic Canada and an eastern clade found from eastern Greenland to Arctic Russia, in addition to being present in western Greenland and the eastern Canadian Arctic [1]. To do so, we reconstructed a phylogenetic tree including a Pacific walrus mitogenome as outgroup using the maximum-likelihood method as implemented in IQ-tree [44,45]. We visualized this tree using Icy-tree [46] and formatted it with Adobe Illustrator.

### (c) Stable isotopes

*δ*^13^C, *δ*^15^N, *δ*^34^S and non-exchangeable *δ*^2^H were measured on bone collagen. These data might be expected to show differences between walrus populations given: (i) the species' high site fidelity [47] and (ii) inter-regional variability in isotope baseline values depending on factors such as food source, salinity, temperature, sea-ice cover and benthic–pelagic coupling [48–54].

Bone chunks of 0.5–1.1 g from seven of the Kyiv rostra (those also analysed for aDNA) were cleaned by sandblaster, crushed by percussion mortar and de-fatted by ultrasonicating in 8 ml of a 2 : 1 chloroform:methanol mixture (changed every 15 min until the liquid remained clear (≤5 rinses)). The process was then repeated with 8 ml of a 2 : 1 methanol:chloroform mixture. Using the Privat *et al*. [55] protocol, with modifications, the samples were: demineralized in 0.5 M aq. hydrochloric acid at 4°C, gelatinized in a pH 3 aqueous solution for 48 h at 75°C, filtered with Ezeefilters and lyophilized. The samples were not ultrafiltered as this procedure reduces collagen yields and alters amino acid compositions without successfully removing contaminants [56]. Subsamples of the resulting ‘collagen’ were weighed (0.8 ± 0.1 mg) in triplicate into tin capsules and carbon and nitrogen isotope analysis conducted in the Godwin Laboratory, Department of Earth Sciences, University of Cambridge, using an automated Costech elemental analyser coupled in continuous-flow mode to a ThermoFinnigan Delta V isotope-ratio monitoring mass spectrometer. Measurement errors are less than ± 0.2‰ for *δ*^13^C and *δ*^15^N, based on replicate analyses of laboratory and international standards.

Sulfur and hydrogen isotopic analysis, by elemental analyser-isotope ratio mass spectrometry, was performed by Iso-Analytical Limited (Ian Begley pers comm.) on subsamples of the collagen analysed for *δ*^13^C and *δ*^15^N. Procedures followed those described in Barrett *et al*. [3]. The *δ*^2^H values of the collagen samples were provided both as measured (non-exchangeable plus exchangeable hydrogen) and following correction for exchangeable hydrogen by three-point linear calibration using United States Geological Survey standards. Hereafter in this paper, *δ*^2^H refers to non-exchangeable *δ*^2^H.

Stable isotope data and standards are provided in electronic supplementary material, table S2. The data are reported using the international scales Vienna Peedee Belemnite (VPDB) for carbon, air (AIR) for nitrogen [57], Vienna Canyon Diablo Troilite (VCDT) for sulfur and Vienna Standard Mean Oceanic Water (VSMOW) for hydrogen [57,58].

The isotopic results for the Kyiv samples are compared to published data regarding 25 walrus samples with *δ*^13^C, *δ*^15^N, *δ*^2^H and *δ*^34^S measurements. Eighteen of these samples were from traded mediaeval rostra found in Northwestern Europe, 15 of which have been attributed to either the eastern (5) or western (10) genetic clade. The western clade specimens are certain to have arrived in Europe via Norse Greenland, and it is inferred by Barrett *et al*. [3] that four of the five eastern clade examples probably also did so. Four control specimens (two eastern clade and two western clade) were from rostra excavated at the mediaeval site of Igaliku/Gardar in Greenland. The final three previously published specimens represent archaeological control samples from the Barents Sea region: two from Iversfjord in Finnmark and one from Russekeila in Svalbard.

To augment this comparative dataset, seven new control samples were analysed following the protocol noted above. Five were from the tusk sockets of skulls originally collected from a historical kill site (*ca* 1850 CE) on the island of Moffen, Svalbard. Two were from a skull fragment (871–1226 CE) and a pelvis (1226–1500 CE) excavated at Alþingisreitur in Iceland [59]. The five Moffen samples could be sexed based on aDNA and/or bone measurements (electronic supplementary material, table S2). One of the two Icelandic specimens could be sexed based on aDNA (electronic supplementary material, table S2) [60].

Collagen preservation was evaluated for all stable isotope samples [61–64]. For carbon and nitrogen, we considered collagen yield, %C, %N and atomic C/N ratio. Collagen yields ranged from 1.8% to 21.3%. All samples had ≥ 24.0%C and ≥ 8.6%N. All atomic C/N ratios are within the traditionally accepted range for well-preserved collagen (2.9–3.6) and also conform with the more stringent criteria (≤ *ca* 3.35) of Guiry and Szpak [64]. Following van der Sluis *et al*. [52], *δ*^2^H values were accepted for collagen samples which passed the quality assessment criteria used for carbon and nitrogen. For sulfur, atomic C/S ratios of 600 ± 300 and atomic N/S ratios of 200 ± 100 have been proposed as quality control thresholds for mammals [63]. Only samples falling within these ranges are included in the present study.

Additional stable isotope measurements have been published for bone collagen from Atlantic walrus (e.g. [53,65]), but in no other case are there matching values for *δ*^13^C, *δ*^15^N, *δ*^2^H and *δ*^34^S that satisfy all of the above-mentioned quality control criteria. Comparison with data from known mediaeval rostra found in Western Europe is ideal; it offers the potential to evaluate whether or not the mediaeval eastern European examples from Kyiv are likely to have derived from the same sources (most Western European examples have previously been interpreted as imports from Norse Greenland [3]). The stable isotope data are compared using t-tests (with Holm correction for multiple tests) and principal component analysis (PCA), conducted in R with rstatix 0.0.7 [66] and FactoMineR 2.4, respectively [67]. The number of specimens involved is not conducive to sample classification using machine learning approaches. Specimens falling within the 95% confidence ellipsis (principal components 1 and 2) of stable isotope data from rostra attributed to the western aDNA clade are considered possible candidates for trade via the Norse colony of Greenland.

## 3. Results

**Figure 2.**
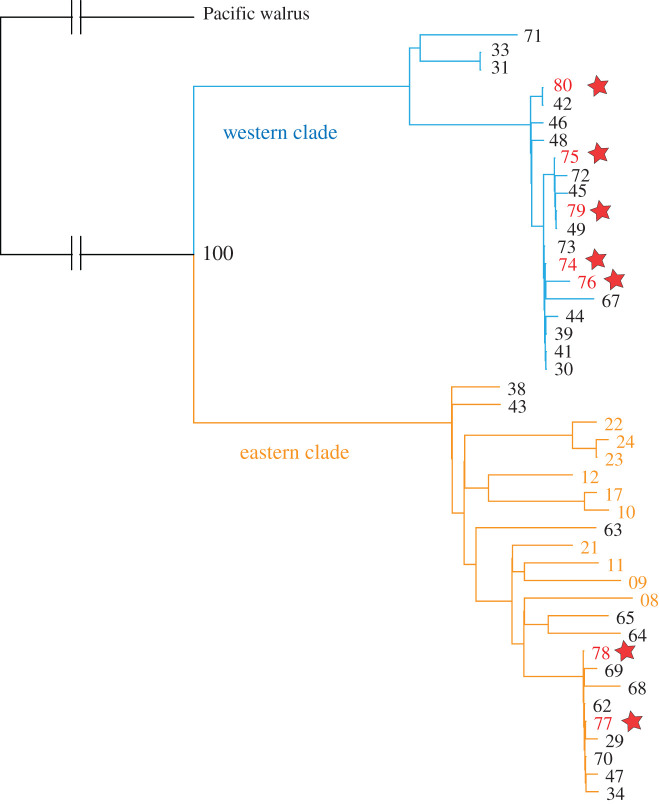
Maximum-likelihood phylogenetic tree of 44 ancient and historic Atlantic walrus specimens. The tree locations of seven specimens from Kyiv (red) are indicated with a star. All control samples from Svalbard (orange) fall into the eastern clade (orange branches). Five specimens from Kyiv fall in the western clade (blue branches) which is geographically restricted to western Greenland and Arctic Canada. The two main clades are significantly distinct (100% statistical bootstrap support). The branch length to the Pacific walrus (here used as an outgroup) is not to scale. Specimen numbers refer to those published by Barrett *et al.* [3] and in electronic supplementary material, table S2, with the omission of the prefix R. (Online version in colour.)

The seven Kyiv rostra specimens studied using stable isotope and aDNA analysis represent separate individual walruses based on osteological criteria and their unique mitochondrial haplotypes (figure 2). Body size and bone portion show that the two other Kyiv fragments are also unlikely to belong to any of the other skulls. Combined osteometric and aDNA evidence indicates that five of the Kyiv rostra represent males and two females (electronic supplementary material, table S2). The two specimens not subjected to aDNA or isotope analysis were too fragmentary to take tusk-socket measurements and are unsexed.

Six of the nine Kyiv rostra exhibit a modification (rough faceting of the tusk sockets) characteristic of types 2 and 3 as described above; one clearly belongs to type 2, with the others being of type 2 or 3 (electronic supplementary material, table S1). A single Kyiv rostrum specimen is a partial tusk socket without faceting; it is therefore probably of type 1. The remaining two Kyiv finds are too fragmentary to elucidate how they were or were not sculpted.

A maximum-likelihood tree based on aDNA of the Kyiv rostra places five specimens (one of type 2, three of type 2/3 and one of uncertain type) in the western clade and two (one of type 2/3 and one probably of type 1) in the eastern clade (figure 2; electronic supplementary material, figure S2 and table S2).

The Kyiv samples have narrow ranges of stable isotope values: −12.7 to −13.6 for *δ*^13^C, 10.4 to 11.8 for *δ*^15^N, 13.5 to 14.6 for *δ*^34^S and 1.2 to 39.0 for *δ*^2^H. These measurements fall within the ranges of stable isotope data from comparison samples of the western genetic clade (figure 4). When all eastern genetic clade specimens are combined, the Kyiv data also fall within the overall stable isotope ranges of samples from this group (figure 4). However, the eastern clade is widely distributed from Arctic Europe to Greenland and Canada, so comparison at a finer geographical scale is pertinent. The Kyiv samples have significantly higher *δ*^15^N and *δ*^13^C values than control samples from the Barents Sea region (*δ*^15^N: *t* = −4.04, d.f. = 12.70, *p* = 0.003; *δ*^13^C *t* = −3.75, d.f. = 9.89, *p* = 0.008). More generally, samples of the western genetic clade also have higher *δ*^15^N and *δ*^13^C values than those of the Barents Sea (*δ*^15^N: *t* = −5.30, d.f. = 17.4, *p* < 0.001; *δ*^13^C *t* = −3.06, d.f. = 8.77, *p* = 0.014). Partial separation between western clade and Barents Sea walruses is evident in the PCA analysis of the four measured isotopes, with the Kyiv specimens falling within the 95% CI of the western clade. Lastly, the Iceland control samples overlap with the Kyiv stable isotope values in all cases except for *δ*^13^C (figure 4) but are too few (*n* = 2) to be amenable to further statistical analysis.

## 4. Discussion and conclusion

Mediaeval rostra of types 2 and 3 from Greenland and Western Europe have previously been demonstrated to be Greenlandic based on find location and/or attribution to the western aDNA clade [1,3]. Thus, assignment of six Kyiv specimens (five analysed by aDNA and stable isotopes, plus one other) to these types is potentially an indicator of origin. However, the typology of the Kyiv specimens alone is not definitive indication of a Greenlandic source. The same modifications could have been copied elsewhere and other mediaeval Western European rostra assigned to types 2 and 3 are of the widely distributed eastern genetic clade [1,3].

**Figure 3.**
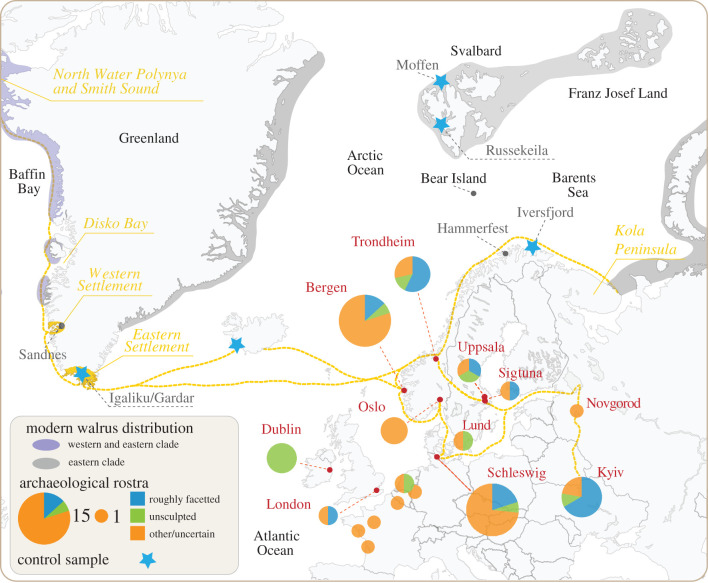
Distribution of mediaeval European finds of walrus rostra, showing the proportion of roughly facetted (type 2 or 3) and unsculpted (type 1) examples in each location. Potential mediaeval trade routes are also shown. Stars mark the locations of control samples from Greenland, Iceland, Finnmark and Svalbard. The modern distributions of the eastern and western genetic clades of Atlantic walrus are shaded. In previous times, walruses were also found as far west as the Hammerfest area in Finnmark, around Iceland, and as far south as the former Western Settlement in western Greenland. Base map after Barrett *et al*. [3] and references therein.

Based on our aDNA evidence and existing knowledge of walrus distributions [1,2,68], the five rostra from Kyiv assigned to the western clade can be confidently attributed to Greenland or the eastern Canadian Arctic (figure 3); they were almost certainly traded via Norse Greenland. The origin of the two eastern clade specimens (R71, R72) cannot be ascertained based on aDNA evidence alone, although eastern clade specimens could also be obtained from western Greenland [1].

**Figure 4.**
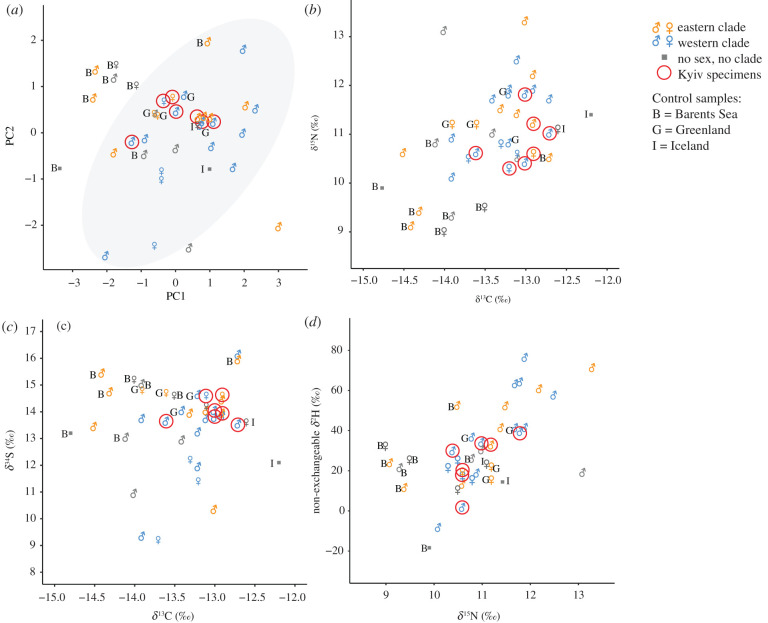
(*a*) Principal component analysis (PC1 and PC2, accounting for 80% of the variance) based on stable isotope values for archaeological walrus rostra and control samples. The variables included are *δ*^13^C, *δ*^15^N, non-exchangeable *δ*^2^H and *δ*^34^S. The 95% CI for all specimens of the western genetic clade is shaded in grey. (*b–d*) Stable isotope values for archaeological walrus rostra and control samples: (*b*) *δ*^13^C and *δ*^15^N, (*c*) *δ*^13^C and *δ*^34^S and (*d*) *δ*^15^N and non-exchangeable *δ*^2^H. Symbols indicate sex and genetic clade where known. For all plots, the Kyiv specimens are circled, and labels indicate the origin of control samples (B is the Barents Sea region, G is Greenland and I is Iceland).

The stable isotope evidence from the seven Kyiv specimens analysed using this method is also compatible with a Greenlandic origin, although not exclusively so. When compared (using PCA) with previously studied finds from Greenland and Western Europe, and control samples collected in the Barents Sea region and Iceland, the stable isotope results of the Kyiv rostra all fall within the 95% confidence ellipsis of western (i.e. Greenlandic/Canadian) aDNA clade samples (figure 4). However, the stable isotope control data do not show complete separation by origin. The two Icelandic control samples also fall within the 95% stable isotope confidence ellipsis of western aDNA clade samples, and two of eight control samples from the Barents Sea region also do so.

In summary, it can be confidently inferred using aDNA, supplemented by typological and stable isotope evidence, that five of the nine Kyiv rostra reached the city via Norse Greenland (R64, R65, R66, R73, R74). With much less certainty, based on typology and/or stable isotopes, three additional specimens may also have done so (R71, R73, R76). There is insufficient evidence to evaluate the origin of the ninth specimen, which could not be studied using aDNA, stable isotopes or typology (R75).

Six of the Kyiv rostra exhibit roughly facetted tusk sockets characteristic of types 2 and 3 (cf. figure 1 and electronic supplementary material, figure S1). This modification is known from Western European hubs, especially Trondheim and Schleswig, where walrus ivory from Norse Greenland was redistributed (figure 3). Both Trondheim and Schleswig peaked as trade centres in the twelfth-century, the date of the Kyiv specimens [69,70]. It may be relevant that Fisher's exact tests show no significant differences in the representation of rostra types between Kyiv and Trondheim (*p* = 1) or Kyiv and Schleswig (*p* = 0.068), whereas comparisons with other mediaeval towns having ≥5 rostra are significant (Dublin *p* = 0.024; Bergen *p* = 0.039) (*p*-values incorporate Holm corrections for multiple tests). From Trondheim, rostra could have been shipped south along a coastal route to Schleswig or east to Sigtuna via a longstanding overland trade route [7,71]. Both Schleswig and Sigtuna have produced distinctive artefacts (e.g. ceramic resurrection eggs) showing trade links with Kyiv and Novgorod [72]. From Schleswig and Sigtuna, trade to Eastern Europe followed the Gulf of Finland and Lake Ladoga. The Rivers Volkhov and Dnieper then led merchants to Novgorod and Kyiv. Although only one mediaeval rostrum is known from Novgorod, this city has produced much twelfth-century walrus ivory [9,10].

Unlike Novgorod, excavations in Kyiv have thus far yielded only a few walrus *ivory* objects [11,12]. Yet, tusks were clearly extracted on the riverside at or near the 35 Spaska Street site in the Podil district. This part of the lower town served as a market and manufacturing area. It was situated on flat land between the Pochaina and Dnieper rivers on the one hand, and the Kyiv Hills on the other. The Pochaina creek provided a natural harbour, and thus Podil was one of the first districts of the city to develop, with craft workshops, a trade area, churches, cemeteries and dwellings. The Spaska excavation was near the former harbour, being located very close to the mouth of the no-longer extant Pochaina. Horizons 5 and 6, where the walrus rostra were excavated, revealed many imported artefacts of mid to late twelfth-century date, from both north (e.g. Germany) and south (e.g. Byzantium) [20–22].

It is likely that walrus ivory was prepared for onward trade at 35 Spaska. The cultural and trade connections of mediaeval Kyiv are typically discussed in terms of strong links with Byzantium [23]. This trade included some walrus ivory, based on archaeological and historical evidence; examples are a gaming piece from Silistra, Bulgaria, and a mid-twelfth-century Byzantine reference to an ink stand (attributed to Rus' craftmanship) made of ‘fish bone', probably walrus tusk [16,73]. Most Byzantine ivories were instead of elephant tusk [74], but, as discussed above, ‘fish teeth' (walrus tusks) were highly valued in Western and Central Asia. The earliest, tenth- and eleventh-century, Arabic authorities to mention the trade of ‘fish teeth' to Asia (e.g. al-Muqaddas̄ı and al-B̄ırūn̄ı) refer to the intermediary role of Bulghār on the River Volga [14,15,75], rather than the Kyivan Rus' on the Dnieper. However, Viking Age trade between Scandinavia and the Volga declined in the eleventh century [15,76]. Based on the rostra finds reported here, it is reasonable to hypothesize that a Dnieper route may have augmented or replaced pre-existing practices. Little can yet be said of the origin of earlier ‘fish teeth' traded along the Volga. Nevertheless, it may not be a coincidence that the earliest reliably dated account is thought to be that of al-Muqaddas̄ı attributed to 985–990 CE [75,77]; Norse Greenland was settled *ca* 985 CE [8]. We can now hypothesize that—although traded through Russia and Ukraine—many of these ‘fish teeth' may also have ultimately been from Greenland.

Our observations indicate that demand for the products of Norse Greenland's walrus hunt stretched (counterintuitively, given closer populations in the Barents and Kara Seas [78,79]) to Ukraine and perhaps also onward to Byzantium and Asia. This discovery helps explain previously published evidence for depletion of the Greenlandic walrus population during the years of Norse occupation [3]. Study of mediaeval rostra from Western Europe has shown that by the thirteenth to fourteenth centuries CE, smaller (more often female) walruses were harvested—increasingly of a genetic clade that is most common in the northernmost area of western Greenland and in the eastern Canadian Arctic [3]. Moreover, Norse artefacts from the thirteenth to fourteenth centuries have been found in the extreme north, as far as Smith Sound and Ellesmere Island [80–82], implying the need for increasingly long-range hunting and/or trading with the indigenous peoples of the Arctic.

The Kyiv rostra pre-date this evidence for serial depletion and the sex ratio (five males, two females) is consistent with the preference for large male walruses prior to the thirteenth to fourteenth centuries. Yet the finds are evidence of an expanding demand for Greenland's walruses that drove a wildlife trade with widespread consequences. These consequences (e.g. the viability of the Norse colony of Greenland [3]) were felt by hunted and hunters, traders and townspeople, artisans and patrons, along extensive networks stretching from the High Arctic to the banks of the Dnieper and beyond. The Kyiv walruses illuminate the surprising antiquity of ecological globalization on an intercontinental scale and the pertinence of historical ecology research to understanding wildlife population history and human socio-ecological systems alike.

## Data Availability

Archaeological, osteometric and stable isotope data for the walrus specimens analysed here are provided as electronic supplementary material, tables S1 and S2 [83]. Summary aDNA data are provided as electronic supplementary material (electronic supplementary material, table S2) [83], and raw sequence data are available from the European Nucleotide Archive (ENA) with the following accession numbers: PRJEB25536 and PRJEB48103.
